# On the Use of Rhatany in the Treatment of Fissure of the Anus, Prolapsus Ani, Leucorrhœa, and Some Other Affections—with Cases

**Published:** 1841-05-08

**Authors:** W. Poyntell Johnston, J. B. Biddle


### MEDICAL EXAMINER.

DEVOTED TO MEDICINE, SURGERY, AND THE COLLATERAL SCIENCES.

No. 19.] PHILADELPHIA, SATURDAY, MAY 8, 1841. [Vol. IV.

### ORIGINAL COMMUNICATIONS.

*On the use of Rhatany in the treatment of Fissure of the Anus, Prolapsus Ani, Leucorrhœa, and some other Affections—with Cases.* By W. Poyntell Johnston, M. D., and J. B. Biddle, M. D.

In the thirty-fifth number of the third volume of the Medical Examiner, in a letter from Professor Martins of Paris, the attention of the profession was called to the employment of rhatany in the treatment of fissure of the anus, at the suggestion of M. Bretonneau, of Tours. Cases were cited in which this remedy was used with the happiest effects by MM. Bretonneau, Trousseau, and Marjolin, in the troublesome affection mentioned. The formula recommended by M. Trousseau was a combination of the tincture and extract of krameria. Since the publication of this paper, we have resorted to the astringent in question, not only in the management of fissure of the anus, but also in cases of prolapsus ani, leucorrhœa, and some other affections, with results entirely confirmatory of the statements of our correspondent as to the remarkable effects of the remedy.

*Cases of Fissure of the Rectum.* By W. P. Johnston, M. D.

The first case which presented itself after the publication referred to was a physician from Virginia, who consulted me for an affection which he regarded as hæmorrhoids, conjoined with a prolapsus of the anus. Upon examination I found a deep fissure of the anterior and left side of the rectum, about an inch and a half in length, extending through the entire thickness of the mucous membrane, and exposing the muscular coat of the intestine. This was accompanied by a prolapsus when he strained. I determined to employ the rhatany, as recommended by our correspondent; and requested my patient, an intelligent physician himself, to keep notes of the case; these notes he has furnished me in the following letter:

"Philadelphia, Jan. 31st, 1841.

"*Dr. W. Poyntell Johnston:*

"Dear Sir,—The first signs of this disease made their appearance in the summer of 1839, and came on in this way:—I found that whenever I had an evacuation from my bowels, that my stools were always attended with more or less pain, and not unfrequently with a discharge of blood. Taking now much exercise on horseback, I thought that I must have a slight attack of hæmorrhoids; and, upon consulting with a physician, he, without making any examination, came to the same conclusion, and accordingly I was put under treatment for this disease; but, having used for some time, without any relief, the remedies usually prescribed for this complaint, I abandoned them, and determined to do nothing more than keep my bowels in a soluble condition, and avoid all articles of diet of an irritating and stimulating nature, hoping that, by this course, I might effect a cure. But finding that my disease was making rapid progress—for every evacuation, especially if my bowels were at all constipated, was attended with severe pain, and followed by a disagreeable, burning sensation, which continued frequently for hours—and seeing, too, that my general health was suffering—for my whole digestive apparatus was much out of order—I determined to subject my rectum to an examination, and to follow rigidly any treatment that would be calculated to relieve me. Accordingly, I applied to you for medical advice; and, much to my surprise, was informed that, instead of piles, I was labouring under fissure of the rectum—a disease which I had always been under the impression could only be cured by a section of the sphincter ani muscle. For this disease I was requested by you to use the following enema night and morning:—Ext. Rhatany, ℨjss.; Tinct. Rhatany, ℨss.; Water, Oss. Before using this enema I was requested to inject with half a pint of flaxseed or barley water, which was to be retained about half an hour,—and then, after the lapse of twenty or thirty minutes, to throw up the rhatany injection, which was to be retained as long as possible. I was also requested to abstain from all articles of food of a stimulating nature.

"This treatment I strictly followed—the enema at different times being increased in strength—and from the very time I commenced with this remedy, my sufferings began to abate; and I am happy to say that I am now entirely restored to my former good health, having used the medicine, in the way described, at intervals during four months, which was some time after every vestige of the disease had disappeared. Yours, &c."

The fissure was entirely cicatrized in less than three weeks. The injection of cold water, as recommended by M. Bretonneau, was found so severe that I discontinued it on the second day, and substituted tepid flaxseed or barley water. The mixture of the tincture with a solution of the extract of rhatany, is by no means an elegant preparation, and the precipitate which forms is occasionally highly irritating; I discontinued it towards the termination of the above case, and substituted the following, which is far more neat, and equally efficacious.

℞. Pulv. Rad. Krameriæ, ℥iij.

Aqua Font., ℥vj.

Pass the water six times through the rhatany in a displacement apparatus, and give it as an injection. Each ounce contains about twenty grains of extract. This formula was found to answer so admirably that we have since employed no other.

In the month of January I was consulted by a gentleman of this city, aged fifty, who for many years had suffered much distress at every evacuation. He had been repeatedly treated for hæmorrhoids with but little benefit. Upon examination I discovered two fissures of the rectum—one towards the perineum, deep, painful, and bleeding on the slightest touch—the other towards the coccyx, was more superficial. I directed him to wash out the rectum, every morning, with half a pint of tepid barley water, to be followed after a lapse of twenty minutes by the injection of rhatany. I directed him also to repeat the latter injection without the barley water, every night, and to retain it, if possible, till the morning. He experienced immediate relief; the distressing symptoms disappeared; and although on two separate occasions the almost cicatrized fissures were broken open by a costive stool, the result of indulgence at the table, and a neglect to use his daily injection,—yet, at the end of two months a cure was complete, and the parts have continued in a perfectly healthy state up to this date.

A third case of fissure of the rectum, for which I was consulted in the latter part of April, is still under treatment; the same plan is followed as detailed above, with every prospect of entire success.

But it is not only in fissure of the rectum that the rhatany will be found so serviceable: we have employed it with equally marked effects in prolapsus ani, in leucorrhœa, and in one instance, as a gargle in a spongy and ulcerated condition of the lining membrane of the mouth.

*Case of Prolapsus Ani of three years' standing, cured by two injections of the Rhatany.* By J. B. Biddle, M. D.

When called to the subject of this case, a young female, I found her suffering from a violent attack of hysteria, brought on by the descent of the bowel, which she had not been able for some time to reduce. I was informed that she had latterly been subject to these attacks upon the descent of the bowel, when she was unable for any length of time to restore it—not, however, previously of the severity of the present fit.

She had been suffering from prolapsus for three years past, gradually increasing in character, until the bowel now descended about three inches. Upon the restoration of the bowel, and the administration of palliatives, the attack of hysteria subsided. For the radical cure of the exciting cause, I proposed the use of injections of the infusion of rhatany, prepared after the method before mentioned. The patient was directed to evacuate the bowels with a flaxseed mucilage injection, and afterwards to throw in the rectum about six ounces of the rhatany infusion, making efforts to retain it. The next day she did not suffer from a recurrence of the prolapsus, and, indeed, after repeating the injection the subsequent evening, she felt so confident of permanent relief, that she desisted from further resort to the remedy, and has, since, a period of six weeks, remained permanently cured. The effects of the rhatany, in this instance, it cannot be disputed, were marked and decided.

*Case of severe Leucorrhea of four years' standing, cured in two weeks, by the use of injections of Rhatany.* By J. B. Biddle, M. D.

This patient was a young married woman, twenty years of age, who had suffered from leucorrhea from the age of puberty. She had at the time she came under my care, an infant five months old. She was at this time suffering from leucorrhea in a very distressing form. She had two ulcers upon the labia, and a bubo on each groin just upon the point of suppurating, and suffered very much in urinating. The circumstances of the case render it quite certain that these symptoms were not other than the results of the excoriating discharge. Her general health was impaired, digestion deranged, loss of appetite, with febricules at night. Enjoining rest, and the constant use of mucilaginous applications, I directed an injection of rhatany to be used twice a-day; at first in combination with a demulcent infusion, and finally of full strength. This was very soon followed by improvement, and, at the end of two weeks, the patient pronounced herself quite free from the discharge. No difficulty was experienced with the secondary symptoms, and, under the use of the hydriodate of iron, and generous diet, her general health has become entirely restored. With any of the long list of astringent injections, recommended for the cure of leucorrhea, I think the rhatany may fairly challenge competition.

