# Errata

**Published:** 1841-05-29

**Authors:** 


*Errata.*—In the paper by Drs. Johnston and Biddle, on the use of rhatany in the treatment of fissure of the anus, leucorrhœa, prolapsus ani, &c., it was erroneously stated that three ounces of rhatany, and six ounces of water, were required for each injection. We are glad to learn from Mr. Oliver, by whom the infusion was prepared, that one-half this amount of root suffices completely to saturate the fluid, and that the employment of more is a mere waste of material. We have received the following note upon the subject.

